# Respiratory syncytial virus: from pathogenesis to potential therapeutic strategies

**DOI:** 10.7150/ijbs.64762

**Published:** 2021-09-27

**Authors:** Zifang Shang, Shuguang Tan, Dongli Ma

**Affiliations:** 1 Institute of Pediatrics, Shenzhen Children's Hospital, 518026 Shenzhen, Guangdong Province, China.; 2 CAS Key Laboratory of Pathogenic Microbiology and Immunology, Institute of Microbiology, Chinese Academy of Sciences, 100101Beijing, China.

**Keywords:** Respiratory syncytial virus, Pathogenesis, Infection, Intervention, Antibody, Vaccine

## Abstract

Respiratory syncytial virus (RSV) is one of the most important viral pathogens causing respiratory tract infection in infants, the elderly and people with poor immune function, which causes a huge disease burden worldwide every year. It has been more than 60 years since RSV was discovered, and the palivizumab monoclonal antibody, the only approved specific treatment, is limited to use for passive immunoprophylaxis in high-risk infants; no other intervention has been approved to date. However, in the past decade, substantial progress has been made in characterizing the structure and function of RSV components, their interactions with host surface molecules, and the host innate and adaptive immune response to infection. In addition, basic and important findings have also piqued widespread interest among researchers and pharmaceutical companies searching for effective interventions for RSV infection. A large number of promising monoclonal antibodies and inhibitors have been screened, and new vaccine candidates have been designed for clinical evaluation. In this review, we first briefly introduce the structural composition, host cell surface receptors and life cycle of RSV virions. Then, we discuss the latest findings related to the pathogenesis of RSV. We also focus on the latest clinical progress in the prevention and treatment of RSV infection through the development of monoclonal antibodies, vaccines and small-molecule inhibitors. Finally, we look forward to the prospects and challenges of future RSV research and clinical intervention.

## Introduction

RSV was first isolated from chimpanzees with respiratory diseases in 1955 1. In 1957, the virus was isolated from babies with severe lower respiratory tract diseases 2. Since then, RSV has been proven to be a ubiquitous pathogen, causing a great burden of disease on children, the elderly and high-risk adults. In particular, almost all children younger than 2 years old will be infected, and one-half of these children will be infected twice during this period 3. Globally, RSV causes approximately 60,000 deaths of hospitalized children younger than 5 years old each year 4. It is the leading cause of infant hospitalization in the world and the second leading cause of infant death after malaria. Over the years, scientists' continued awareness of the serious threat posed by respiratory syncytial virus has inspired researchers, including those in pharmaceutical companies, to develop effective interventions.

RSV is a polymorphic negative sense, single-stranded RNA virus belonging to the *Orthopneumovirus* genus of the family *Pneumoviridae* in the order *Mononegavirales* 5. The virus is mainly transmitted by close contact with saliva or mucus droplets. After the virus replicates in the epithelial cells of the nasopharynx and upper respiratory tract for a short time, the released virus particles may transfer to the bronchioles or alveoli of the lower respiratory tract. The immune response in patients infected with RSV causes neutrophils to infiltrate and narrow the airways, leading to respiratory diseases such as bronchiolitis. In general, approximately 3 to 7 days after being infected, patients begin to develop some common symptoms, including fever, runny nose, stuffy nose, cough, and chest tightness 6.

**Figure 1:**
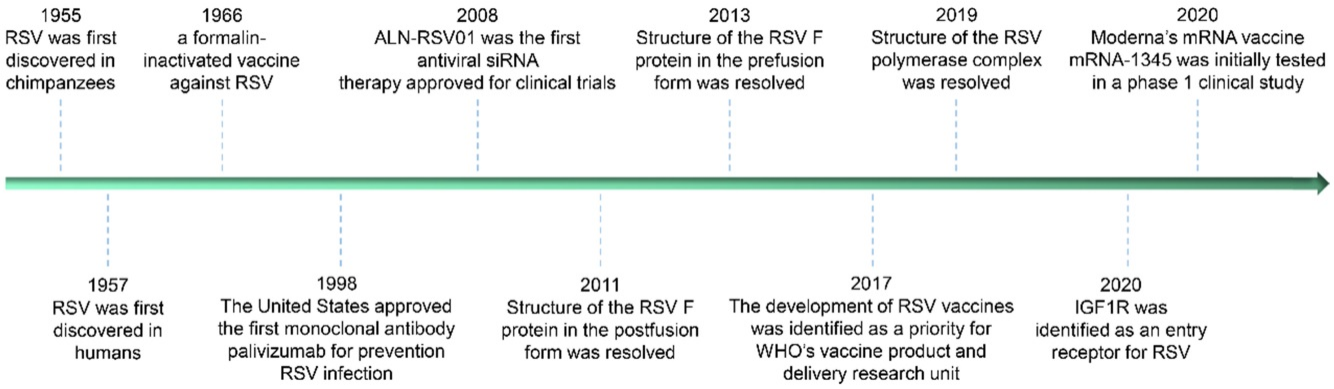
Timeline of RSV discovery and important related events.

Currently, there is no effective means for the prevention and treatment of RSV infection has been approved, but in the past decade, dozens of candidates for the prevention and treatment of RSV diseases have been screened under great continuous efforts (particularly in structural biology and single B cell techniques). More than 30 different candidate vaccines and more than 10 kinds of antibodies are in clinical or preclinical development. These promising prevention and treatment methods will effectively improve some deleterious effects of infection, such as vaccine-enhanced diseases caused by RSV infection in the early stage and the shortened half-life of RSV antibodies in the human body. Some of the iconic events in the study against RSV infection since RSV discovery are listed in Figure 1 below.

In this review, we briefly describe the structure of RSV virions, the latest progress in understanding host surface virus-binding receptors and the viral life cycle. Then, we discuss recent advances in understanding the pathogenesis of RSV infection. We also highlight the latest progress in the prevention and treatment of RSV infection, including the development of vaccines, monoclonal antibodies and small-molecule fusion inhibitors. Finally, we look forward to the prospects of RSV research and clinical intervention in the future.

## Genome structure, entry and the life cycle

### The virion genome structure

Human RSV is mainly composed of two subtypes (A and B), which belong to the *Orthopneumovirus* genus in the *Pneumoviridae* family and *Mononegavirales* order, and is prone to genetic changes. In terms of structural morphology, RSV is a pleomorphic virus particle in which the filovirus is the dominant form, with a diameter of approximately 50 nm and a length from 1 to 10 μm, while the spherical virus particle is generally from 150 to 250 nm in diameter 5.

**Figure 2:**
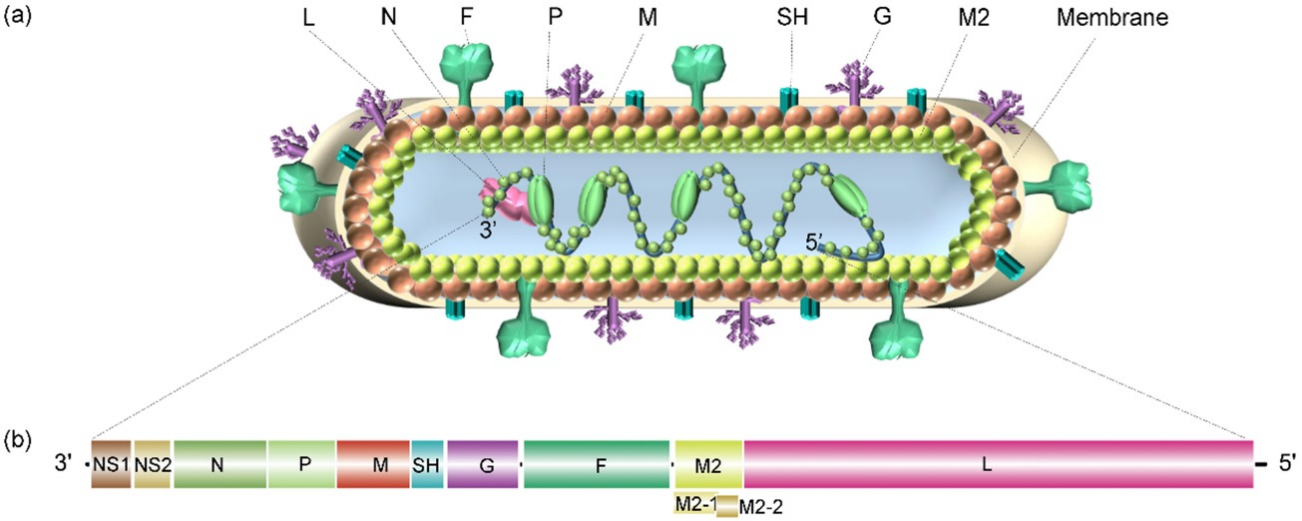
** Schematic diagram of the RSV virion and its genome structure. (a)** The general structure of the RSV virion and its encoded proteins. **(b)** The genome organization of RSV consists of 11 open reading frames (ORFs), including 2 ORFs adjacent to the 3′ leader region that encode nonstructural proteins related to evading the innate immune response, and ORFs that encode structural proteins: nucleoprotein (N), phosphoprotein (P), matrix protein (M), small hydrophobic (SH) protein, glycoprotein (G), fusion protein (F), M2 protein, and polymerase (L) protein.

The virion contains an unsegmented, single-stranded, antisense viral RNA genome of approximately 15.2 kb. The full-length genome is segmented into 10 genes encoding 2 nonstructural proteins and 9 structural proteins (a total of 11 proteins; Figure 2). The nonstructural proteins NS1 and NS2 are primarily described as being related to evasion from the innate immune response, in which the unique structural region of NS1 is involved in the regulation of the host response, including inhibition of the type I interferon (IFN) response, inhibition of dendritic cell maturation and promotion of the inflammatory response 7. NS2 can bind and inhibit the ubiquitination of inactive forms of retinoic acid-inducible gene-I (RIG-I) and melanoma differentiation-associated protein 5 (MDA5) and prevent the production of downstream signals and type I IFN 8.

The nucleoprotein (N) is critical for tight viral binding to genomic RNA. Phosphoprotein (P) is an important polymerase cofactor that forms tetramers 9, which acts not only as a cofactor of N protein monomers and connects the L protein to the nucleoprotein-ribonucleic acid complex 10 but also as a chaperone protein to prevent the binding of newly synthesized N protein to host cell RNA 11. The matrix protein (M) is located inside the viral envelope and plays a role in supporting the viral envelope. Additionally, it participates in the transcription process of viral RNA. The small hydrophobic (SH) protein is a pentameric ion channel that is thought to be related to the delayed apoptosis of infected cells 12. Glycoprotein (G) is rich in serine and threonine (~30-35%) and proline residues (~8-10%). After translation, this viral glycoprotein is modified into a highly glycosylated protein with 4-5 N- and 30-40 O-linked sugars, which account for approximately 60% of the molecular weight of these mature glycoproteins 13. As an attachment protein, this glycoprotein connects virions to target cells by interacting with host cell surface molecules. In addition to the membrane-bound form, this viral G protein is also produced in a secretory soluble form upon alternative translation of the second AUG codon (M48) in the ORF, which is located in the TM domain. Then, the N-terminus is hydrolyzed and modified to form a new N-terminus. Although both the membrane and secretory forms mediate the immune escape of RSV, the soluble form also induces the production of specific antibodies targeting the viral G protein and reduces the antiviral activity of leukocytes that are mediated by the fragment crystallizable region (Fc) 14,15. The G protein is disordered and consists of an N-terminal hydrophobic transmembrane domain (approximately 40-65 amino acids) and a C-terminal extracellular domain (approximately 66-298 amino acids). There is a short central conserved region (CCR) in the extracellular domain of viral G, which is sandwiched between two “mucin-like” regions (determining the antigenic subgroups A and B) flanking both sides of the protein 16. The CCR contains a cystine knot and four cysteines that form two disulfide bonds with 1-4 and 2-3 topologies 17,18. Altogether, viral G has a high degree of sequence variation within and between virus species. Fusion protein (F) is synthesized into a precursor (F0) containing 574 amino acids, which is then cleaved by a host cell protease to produce a heterodimer connected by disulfide bonds formed between F1 and F2 subunits accompanied by the release of the p27 peptide. Then, a mature F protein trimer form is formed to mediate the fusion of the virus membrane and the host cell membrane 19. In addition, the F protein is also critical for the fusion of infected cells with neighboring cells, thus forming a unique syncytium. The M2 gene has two overlapping ORFs producing the M2-1 and M2-2 protein. The former is a factor involved in the transcription process 20, and the latter is a protein that regulates the transition from transcription to genome replication. The L protein contains three conserved enzymatic domains: an RNA-dependent RNA polymerase (RdRp) domain, a polyribonucleotide transferase (PRNTase or capping) domain and a methyltransferase (MTase) domain, which catalyzes cap methylation 9. RdRp initiates two RNA synthesis processes induced by viral promoters: genomic replication at position 1U and mRNA transcription at position 3C. A single promoter can be initiated directly from positions 1U and 3C independently of each other, and the same RdRp can accurately select both of these sites. RdRp tends to start at 3C, but the selection of initiation sites may be regulated by the relative concentrations of ATP and GTP 21.

### Entry of RSV and its host binding receptors

**Figure 3:**
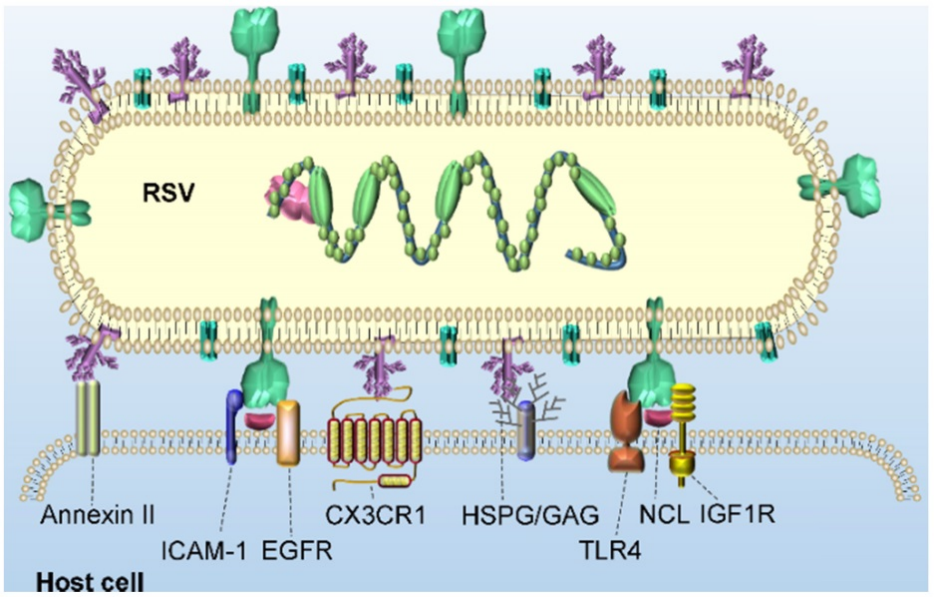
** Potential receptors of RSV binding for virus entry into host cells.** Potential receptors, such as annexin II, HSPG/GAG and CX3CR1, bind to the RSV G glycoprotein, and TLR4, ICAM-1, EGFR, NCL and IGF1R bind to the RSV F glycoprotein, tether RSV virions to the cell surface.

The process by which RSV virions enter host cells is primarily initiated by the binding of virions to the surface molecules of host cells and the fusion of the virus and host cell membranes. As mentioned in the previous section, three structural proteins are encoded to localize to the surface of the virus: SH, G and F. The RSV SH protein is not necessary for *in vitro* infection 22. Therefore, in the following sections, we focus on substantial advances in the understanding of the interaction of RSV G and F proteins with a host cell. Figure 3 shows a schematic diagram of RSV binding to potential receptors in host cells.

The main function of the viral G protein is to bind virions to the cell surface by interacting with host cell adhesion molecules. Annexin II is a peripheral membrane protein expressed on endothelial cells in a variety of tissues and organs that participates in many activities located on the cell surface. For example, annexin II binds to phospholipids on the surface of EGTA-treated endothelial cells with high affinity in a calcium-dependent manner. On the surface of metastatic lymphoma cells, annexin II seems to enhance the cancer cells adhesion to hepatic sinusoid endothelial cells 23. In addition, the extracellular matrix component tenascin C strongly binds to annexin II on gliomas and endothelial cells and initiates migration, cell proliferation and loss of focal adhesion 24. As a potential RSV receptor, annexin II has been found to bind to the RSV G protein in Hep2 cells, which can be inhibited by the selectin antagonist TBC1269 25. Heparan sulfate proteoglycan (HSPG) and other glycosaminoglycans (GAGs) on the surface of host cells seem to promote the infection of RSV in immortalized cell lines, which may be realized by the promoted binding of positively charged residues in the heparin-binding domain (HBD) of the RSV G protein 26. Notably, in infected Hep-2 cells, the molecular weight of the G protein expressed by an RSV virion is 95 kDa after glycosylation, and the molecular weight of its polypeptide skeleton is 32 kDa. However, a G protein with higher glycosylation levels and a greater molecular weight, 170 kDa, was produced in HBE culture, in which virions of both laboratory-adapted strains and clinical isolates of RSV subgroups A and B were evident 27. However, the absence of HSPG on the surface of human ciliated airway epithelial cells greatly eliminated the interaction between G and HSPG in these cells 28. In contrast, CX3C chemokine receptor 1 (CX3CR1) on the apical surface of ciliated cells in human airway epithelial (HAE) culture binds to CX3CL1 and mediates its adhesion and migration. A study confirmed the interaction between host CX3CR1 and RSV G. A non-neutralizing monoclonal antibody (mAb) against the CX3C motif of the viral G protein could inhibit the effective infection of RSV in culture. In addition, CX3CR1-deficient mice were significantly less susceptible to RSV infection than CX3CR1-expressing mice 29.

Another major glycoprotein on the surface of RSV is the F protein, which is a class I fusion protein. The F protein is anchored on the surface of the RSV membrane by a transmembrane domain and is a 'spring-loaded' trimer. Extensive and in-depth studies have been carried out on the binding receptors of the F protein. Toll-like receptor 4 (TLR4), a pattern recognition receptor (PRR) member of the Toll-like receptor family, is known as a sensitive receptor of gram-negative lipopolysaccharide (LPS). It has been shown to mediate the activation of the intracellular NF-κB signaling pathway and inflammatory cytokines to trigger the innate immune system 30. RSV replicating in higher concentrations in TLR4-deficient mice persisted longer than that replicating in normal mice. This finding suggests that the expression of TLR4 plays an important role in controlling RSV replication *in vivo* and mediates the innate immune response of monocytes to produce IL-6 upon exposure to the RSV F protein 31. Intercellular adhesion molecule-1 (ICAM-1), a type 1 glycoprotein in the immunoglobulin superfamily, promotes the entry and infection of RSV in human epithelial cells by binding to RSV F protein, which is important for viral replication and infection 32. In addition, it has been reported that epidermal growth factor receptor (EGFR) expressed on the apical surface of differentiated bronchial epithelial cells can interact with the RSV F protein and promote fusion of host-virus membrane 33. Tayyari *et al.* found that RSV interacted with nucleolin (NCL) of host cells via the F protein and specifically bound to NCL on the surface of apical cells *in vitro*. Confocal microscopy showed that NCL and RSV virions were colocalized at the surface of the cultured cells. When these cells were preincubated with NCL-specific antibodies, the colocalization of RSV and NCL proteins on the cell surface decreased significantly 34. Another study found that the levels of NCL and TLR4 colocalized with the F protein increased in the early stage of infection and then decreased. Although NCL is the most widely studied of the potential receptors described above, it has also been shown to interact with many other viruses, including HIV-1 35, human parainfluenza virus type 3 36, enterovirus71 37, human influenza A 38 and rabbit hemorrhagic disease virus 39. These findings seem to suggest that NCL may act as a cofactor of the RSV F protein not as a major binding receptor. Most recently, Griffiths *et al.* discovered insulin-like growth factor 1 receptor (IGF1R) as a novel receptor of RSV 40, which may provide a new insight for the entry of RSV into the host.

An endocytic entry mechanism is initiated of RSV G protein. RSV infection activates ATPase Na^+^/K^+^ transporting subunit alpha 1 (ATP1A1) in an RSV G protein-dependent manner, which in turn causes tyrosine kinase c-Src to transactivate EGFR through phosphorylation at EGFR Tyr845 41. The downstream signals of EGFR lead to actin rearrangement, wrinkles on the plasma membrane and phagocytosis of liquid and RSV via macropinosomes upon extension of the plasma membrane. RSV is introduced into large liquid-filled macropinosomes in the form of the viral envelope, which eventually promotes the fusion of RSV and host membranes and RSV entry into the host cell. San-Juan-Vergara *et al.* indicated that in primary NHEB cells, the entry of RSV is caused by the binding of RSV to cholesterol-rich plasma membrane components that promote the semifusion of the RSV envelope and the plasma membrane with complete fusion following in the endosome during endocytosis 26. Krzyzaniak *et al.* believe that the entry of RSV virions involves the interaction between the RSV F protein and EGFR that activates the signal cascade of phosphatidylinositol 3-kinase (PI3K), p21-activated kinase 1 (PAK1) and downstream effectors in host cells. They content that this signaling cascade leads to a series of disturbances, such as actin rearrangement, plasma membrane vesicle formation, and a significant increase in fluid uptake, triggering macrophage-mediated endocytosis. In the macropinosomes in which Rab5 functions, the RSV F protein is cleaved for a second time under the action of an acid-independent furin-like enzyme and ultimately enters the host effectively 42. Recently, Griffiths *et al.* suggested that the binding of the RSV F glycoprotein to IGF1R triggers the activation of protein kinase Cζ (PKC zeta), which then promotes the recruitment of NCL from the nucleus to the plasma membrane, thus enhancing the binding and entry of RSV virions to host cells 40. The related experiments showed that inhibition of PKCζ significantly reduced RSV infection to the same extent as blocking the interaction between RSV and NCL.

### The life cycle of RSV

**Figure 4:**
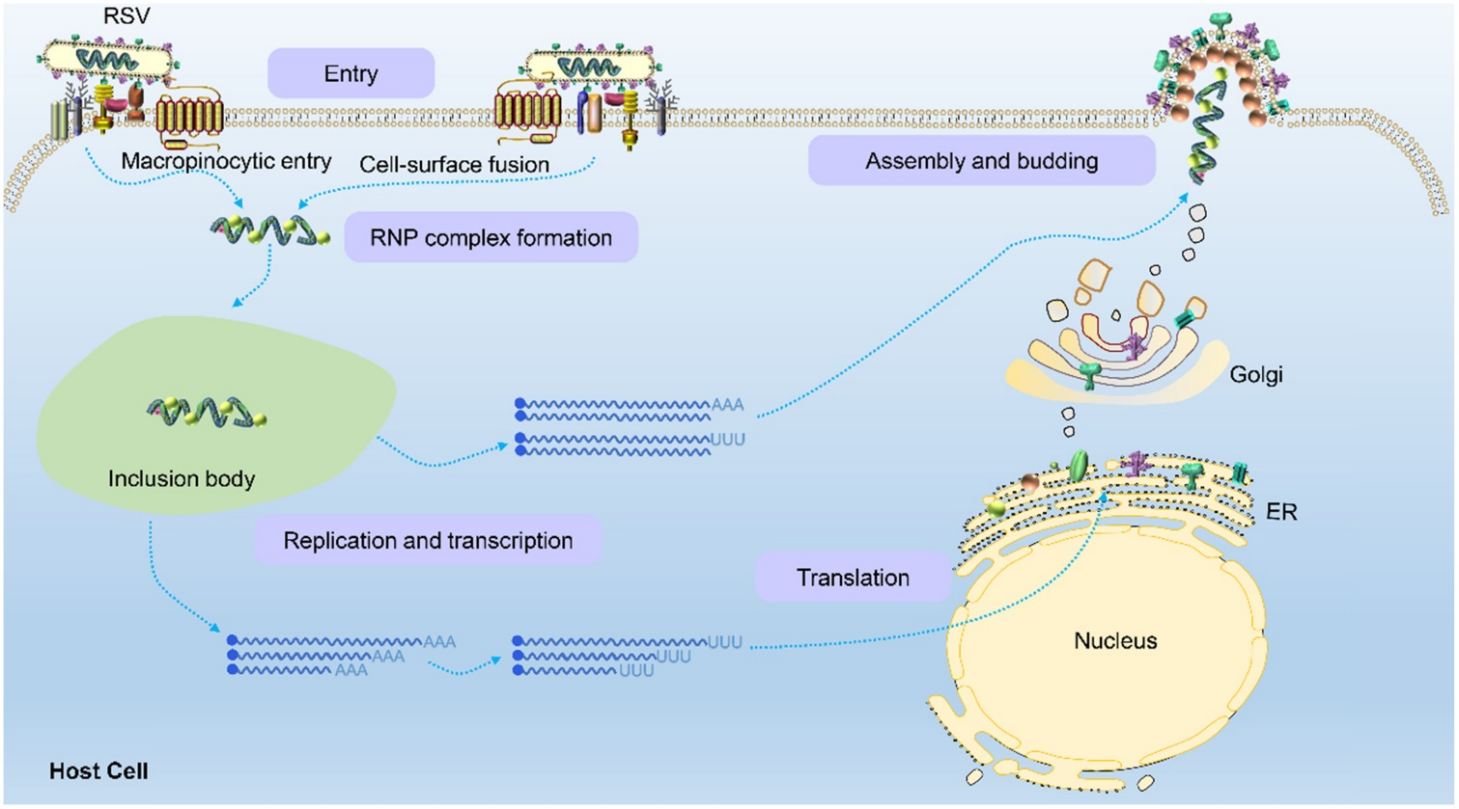
** Schematic of the RSV life cycle.** RSV first enters the host cell via macropinocytosis or cell-surface fusion by binding to the host cell receptor. The virus fuses with the cell membrane and releases the RNP complex to initiate replication and transcription in an inclusion body to produce the genomes, internal component proteins, and surface proteins required by the virus. Genomes are assembled to form new RNP complexes, and proteins are translated on the endoplasmic reticulum and then moved to the Golgi apparatus where they mature. Finally, the RNP complexes are transferred to the plasma membrane to germinate new filamentous RSV virions, completing the viral life cycle.

Once the virion binds and enters a host cell, internal viral components are released. The viral ribonucleoprotein (RNP) complex assembled by L polymerase and viral genomic RNA wrapped by P, M2-1 and N proteins is replicated, transcribed and translated into the various components needed to form viral progeny in a space called the cytoplasmic protein inclusion body (IB) near the intima 43. The IB contains some host proteins, such as the antiviral protein MDA5 44, chaperone protein HSP70 45, poly(A)-binding protein (PABP) and eukaryotic translation initiation factor 4G (EIF4G) 43. Because RSV is a negative sense RNA virus, the RSV genome contains noncoding regions, namely, the leader region and tailer region, at the 3' and 5' ends, respectively. RdRp not only replicates and synthesizes full-length and positive-sense antigenomes but also transcribes viral subgenomic mRNA (Figure 4). Currently, the mechanism by which the polymerase complex switches between replication and transcription has not been elucidated. It has been reported that this switching may be related to the M2-2 protein because its deletion significantly reduced the level of genomic and antigenomic RNA in infected cells 46. Subsequently, the RNP complex is thought to use the trailer region as a promoter to replicate the full-length negative-sense genome at the 3' end of the positive-sense antigenome for the assembly of progeny viruses. In this process of genome transcription, RSV polymerase initially binds a sequence from the polymerase starting point of the leader region (nucleotides 1 to 15) and moves along the RNA genome from the 3' end to the 5' end to produce all 10 subgenomic mRNAs. There are gene start (GS) and gene end (GE) signals on both sides of the template gene region corresponding to each newly synthesized mRNA 47,48. The GS signal guides the polymerase to initiate mRNA synthesis and adds a methylated guanosine cap structure to the 5' end of the newly synthesized mRNA. The GE signal guides the addition of a poly A sequence at the 3' end and induces the release of the mRNA 48. Subsequently, the polymerase continues to slide along the gene sequence after the GE signal until the next GS signal is activated to synthesize the next subgenomic mRNA. Both genomic and antigenomic RNA are directly encapsulated by nuclear protein (N) in the process of synthesis, and each N protein binds 7 nucleotides.

RSV glycoproteins are initially translated into the endoplasmic reticulum and then transported to the Golgi apparatus, where they are glycosylated and rapidly expanded along microtubules to form filaments through dynein-dependent vesicles. The RNP complex loaded with the RSV RNA genome is assembled into filaments after their formation and ultimately transferred to the plasma membrane to sprout new RSV virus particles 49. However, Ke *et al.* performed by cryo-electron tomography to find that the assembly of RSV virions occurred at the plasma membrane 5. When RSV is newly released from infected cells, it is filamentous regardless of virus strain, cell line or the polarization phenotype of the host cell. This characterization is obviously different than that of influenza virus. The morphology of influenza virions is closely related to the polarization phenotype of the host cell and the integrity of its actin microfilaments. When influenza strain A/Udorn/72 is produced in infected polarized epithelial cells, it is filamentous, but in nonpolarized cell types, almost all the virions produced are spherical 50.

## Pathogenesis

RSV is mainly transmitted from person to person through saliva or mucus droplets. Symptoms begin approximately 3 to 7 days after infection with RSV and include fever, runny or stuffy nose, cough, chest tightness, wheezing and dyspnea 6. Notably, when an infected person coughs or sneezes, vesicles from extracellular amoebae, among the most common organisms in relatively humid environments, may contribute to the persistence of respiratory viruses in the environment 51. RSV infection can induce microtubule-/dynein-dependent mitochondria to gather around the nucleus and translocate to the center of the microtubule tissue 52. These changes lead to impairment of mitochondrial respiratory function, loss of mitochondrial membrane potential and elevation of mitochondrial reactive oxygen species (ROS), which in turn increase the replication and titer of RSV. In addition, RSV infection can stabilize the expression of hypoxia-inducible factor-1α (HIF-1α) in infected cells, which profoundly changes cell metabolism to facilitate glycolysis and the pentose phosphate pathway activation and further enhance the replication ability of RSV 53.

RSV infection is most likely to affect the respiratory system, with most of the damage to the airway mediated by the immune response, not by the virus replication itself. The main cell to be infected by RSV is the respiratory epithelial cell (AEC). In AEC, RSV F inhibits the production of interferon-λ (IFN-λ) induced by interferon regulatory factor (IRF) 1 (the most critical type III IFN in the antiviral immune response to RSV infection) by inducing EGFR activation, which leads to a continuous increase in viral infection 54. In infected cells, the transcription of viral genes should encode NS1 and NS2 proteins initially, which are essential for host infection, and their function is to inhibit the type Ⅰ interferon (IFN-Ⅰ) response and other components of the immune system. NS1, which has previously been studied more deeply than NS2, is a major participant in immunosuppression. It is reported that NS1 can bind and inhibit various molecules in the signal cascade of IFN-Ⅰ response in retinoic acid-inducible gene I (RIG-I) or Toll-like receptor (TLR) pathway 55. The NS1 and NS2 complexes are transported to mitochondria to form degradosome, which can degrade a variety of proteins in the IFN-Ⅰ pathway 56-58, such as STAT2, TRAF3 (TNF receptor-associated factor 3), TBK1 (TANK-binding kinase 1) and RIG-I. NS1 protein also plays a role in altering CD4^+^/CD8^+^ cells. On the one hand, NS1 inhibits the activation and proliferation of CD103^+^ CD8^+^ T cells, in which CD103 is a molecule that guides CD8^+^ T cells to respiratory mucosal epithelial cells and triggers cytolytic activity 59. At the same time, NS1 also inhibited the activation and proliferation of Th17 cells with antiviral effect. On the other hand, NS1 increased the expression of IL-4 in CD4^+^ T cells and promoted the response of Th2 (T helper cells) by antagonizing IFN-I 59.

IL-33 signaling has been shown to play an important role in airway inflammation caused by RSV infection 60, and neutralizing IL-33 can significantly reduce the occurrence of allergic inflammatory events 61,62. A variety of cell-derived (such as alveolar macrophages and dendric cells) IL-33 can be triggered in the process of RSV infection 60,63, which is thought to depend on the activation of MAPK signaling pathway 64, and then induce the release of nuclear factor-kappa B (NF-κB) and IRF from alveolar macrophages and mast cells during the innate immune response, resulting in the production of IL-6 and tumor necrosis factor-α (TNF-α), which lead to the secretion of tissue mucus. The accumulation of these inflammatory factors further recruits a large number of granulocytes (such as neutrophils) to the infected site. A recent study showed that neutrophils could significantly regulate RSV latent infection and reduce the exacerbation of asthma in children through phagocytosis facilitated by the carcinoembryonic antigen-associated cell adhesion molecule 3 (CEACAM3) protein 65. Recruitment of neutrophils in the airway of patients can increase the expression of some bactericidal proteins, such as myeloperoxidase (MPO) and bactericidal/permeability-increasing protein (BPI) 66. For example, MPO is a powerful bactericidal protein that selectively binds to and quickly kills bacteria such as *Escherichia coli*, *Pseudomonas aeruginosa*, *Staphylococcus aureus* and *Streptococcus pyogenes* in the presence of *Streptococcus pneumoniae* 66. BPI has shown independent antimicrobial activity against gram-negative bacteria, such as *Escherichia coli*, and can neutralize bacterial endotoxins. The antibacterial activity of these bactericidal proteins is helpful in regulating the symbiotic bacteria of the upper respiratory tract.

In addition, IL-33 can increase the expression of thymic stromal lymphopoietin (TLSP) in DC cells and then change the differentiation of T cells to favor CD4^+^ T cells expressing Th2 characteristic cytokines 67. It can also induce type II congenital lymphoid cells (ILC2 cells) the increased secretion of IL-4 and IL-13 levels, which further promote mucus secretion 68. This IL-13-induced Th2 cellular immune response is thought to be closely related to chitinase 3-like 1 protein (CHI3L1); that is, CHI3L1 is proposed to enhance airway hyperreactivity (AHR), inflammatory cell recruitment and mucus production in RSV-infected hosts 69. Finally, excessive mucus secretion and exfoliated airway cilia and airway epithelial cells caused by RSV infection together with neutrophils and lymphocytes in the airway lead to airway obstruction.

A growing body of evidence suggests that severe RSV disease is associated with an inadequate immune response and a low viral load. For example, when the researchers analyzed the upper respiratory viral load and immune response of infants hospitalized with acute bronchiolitis caused by RSV and other viruses, they found that infants with severe disease showed lower RSV viral load and lower concentrations of IFN-γ and CCL5/RANTES compared with infants with moderate disease 70. Heinonen *et al.* drew the same conclusion through transcription mapping and multiparameter flow cytometry combined with a systematic analysis of clinical data. In addition, the latter group found that, compared with severely hospitalized patients, children with mild disease had high expression of plasma cell and inflammatory genes, lower activation of neutrophil and monocyte gene expression, and reduced inhibition of T cell and NK cell gene expression 71. IL-21/IL-21R bound together in the presence of other costimulatory signals drove the differentiation of memory cells, germinal center B cells or plasma cells to produce high-affinity antibodies to protect host cells from pathogens. RSV inhibited the humoral immune response of B cells by negatively regulating the expression of IL-21R on the surface of T follicular helper (TFH) cells and IL-21 in immature B cells. These findings may explain the lack of an immune response 72. In infants with severe respiratory tract infection, the production of IFN-γ by NK cells induced by specific antibodies to RSV was significantly lower than that in uninfected infants, and the activation of these NK cells seemed to be related to Fc fucosylation of RSV-specific antibodies 73.

Notably, during RSV infection, the severity of bronchiolitis and wheezing disease was related to other factors, such as respiratory bacteria. Nasal mucus samples obtained from children with mild and severe RSV diseases were analyzed by 16S ribosomal sequencing to characterize the microbiota 74. The results showed that all five major bacterial communities showed the characteristics of being the dominant bacteria types. RSV infection and hospitalization were positively correlated with an abundance of *Haemophilus influenzae* and *Streptococcus* and negatively correlated with an abundance of *Staphylococcus aureus*. *Streptococcus pneumoniae* (gram positive) and *Haemophilus influenzae* (gram negative) were also found to be the most common bacterial isolates in other studies of lower respiratory tract bacterial coinfections in hospitalized patients with RSV infection 75. These results suggest that airways damaged by RSV infection may be more vulnerable to secondary bacterial infection. The expression of some bacterial receptors, such as intercellular adhesion molecule-1 (ICAM-1), platelet activating factor-receptor (PAF-r) and carcinoembryonic antigen-associated cellular adhesion molecule 1 (CEACAM1), was induced during RSV infection, which enhances the binding of bacteria to prolong lower respiratory tract infection (LRTI).

## Potential intervention strategies

The preclinical and clinical development of RSV intervention can be roughly classified into three categories: monoclonal antibodies, vaccines and small molecules. Each of these strategies has its own unique advantages and disadvantages, but each solution is expected to be a candidate to pass clinical trials.

### Antibodies

Antibodies are important prophylactic treatments for patients at risk of serious RSV infection. RSV virions contain F, G and SH surface proteins, among which the F protein is highly conserved among RSV strains and is the main target of protective neutralizing antibodies. The first treatment used to prevent RSV infection was RespiGam developed by Medimmune, which is a mixture of human intravenous immunoglobulin (IVIG) containing high concentrations of RSV protective antibodies. It is effective in the prevention of severe LRTI caused by RSV infection in high-risk infants. With the introduction of palivizumab (Synagis), Medimmune voluntarily stopped using RespiGam in 2003. Palivizumab is a humanized mouse mAb that can bind to the RSV F protein. It was also the first antiviral mAb approved for human treatment. Palivizumab can effectively prevent RSV hospitalization for high-risk premature infants with pregnancy less than 32 weeks in the first 6 months after discharge 76. However, due to its limited cost and effectiveness, which are similar to those of RespiGam, it is currently approved only for the prevention of RSV in premature infants and infants with cardiopulmonary diseases at birth. Motavizumab (MEDI-524) is a second-generation palivizumab product. It mutates 13 specific amino acid residues located in the variable region of the CDR sequence of the antibody, thus enhancing its affinity and neutralization by 70- and 100-fold, respectively 77. The results from a phase 3 clinical trial revealed that motavizumab showed better neutralization ability and a longer half-life than palivizumab, which makes it a promising candidate. MEDI-557, a next-generation product of motavizumab in which three amino acids are substituted in the Fc region (M252Y/S254T/T256E (YTE)), increased the binding to FcR by 10-fold and increased the serum half-life by 4-fold 78. Two phase I clinical trials on the safety, tolerance and pharmacokinetics of motavizumab have been completed (NCT00578682 and NCT01562938). A clinical trial with healthy adults with nasal RSV infection was suspended due to an unmet schedule and budget (NCT01475305).

Over the past decade, because of the great advances in mAb screening technology, hundreds of human antibodies against RSV F proteins have been isolated and identified. Among these antibodies, many RSV F-specific antibodies have been shown to be much more effective than palivizumab. Kwakkenbos *et al.* transformed peripheral blood memory cells (PBMCs) into germinal center-like B cells that express specific antibodies to RSV by introducing Bcelllymphoma-6 (Bcl-6) and Bcl-xL genes 79. The 50% inhibitory concentration (IC50) of the screened D25 mAb against respiratory syncytial virus A2 virus was 2.1 ng/ml, which was 100-fold higher than that of palivizumab, and the D25 mAb led to significantly reduced function and potency of RSV replication in a cotton rat model. After YTE substitution of three amino acids in the highly conserved Fc region of D25, the nirsevimab (MEDI8897) antibody was generated. The YTE substitution enhanced the binding of IgG1 to the neonatal Fc receptor (FcRN) and improved its anti-degradation performance, thus prolonging the half-life of the antibody in serum (the average half-life ranged from 85 to 117 days) 80. In a phase 1b/2a dose-ranging study (10, 25, and 50 mg of nirsevimab or placebo) with healthy premature infants aged 32-35 weeks, 90% of the inoculated infants exhibited a fourfold increase in serum RSV-neutralizing antibody levels compared with the control group, and the half-life of the antibody supported continuous protection for 5 months 81. The results also showed that nirsevimab significantly reduced the consultation rate and hospitalization rate for RSV infection by 70.1% and 78.4%, respectively, compared with the placebo (saline) control group 82. Currently, a clinical trial on the safety of preventing RSV LRTI in high-risk children is being evaluated (NCT03959488).

Suptavumab (REGN-2222) is a human monoclonal IgG1 antibody specific to the RSV pre-F protein. It is significantly more potent in neutralizing RSV than palivizumab and has good tolerance and a longer half-life in healthy adults 83. However, a phase 3 trial of 1154 premature infants showed that all RSV subtype B isolates had two amino acid mutations in the epitopes, resulting in the loss of suptavumab neutralizing activity, which led to the discontinuation of its clinical development 84. Tang *et al.* screened the potent RSV mAb RB1 from human memory B cells 85. This antibody could bind to a highly conserved epitope on antigenic site IV of the F protein in both RSV subtype A and B. RB1 effectively neutralized a variety of clinical isolates of RSV *in vitro* approximately 100-fold more effectively than palivizumab, and it showed good protective effects in a cotton rat model. Its optimized antibody, MK-1654, is currently being tested in a phase 2b/3 clinical study to evaluate its efficacy and safety in healthy premature and full-term infants (NCT04767373).

In addition, some other pre-F-specific antibodies are also in the preclinical development stage. For example, Orti *et al.* screened a broad-spectrum HRSV- and HMPV-neutralizing antibody MPE8 from the peripheral blood of a donor infected with HRSV and then infected with HMPV by flow cytometry. MPE8 showed great potential not only in preventing HRSV and HMPV infections in newborns but also in immunocompromised adults 86. ALX-0171 is a trivalent RSV-neutralizing nanobody that binds to a palivizumab-like epitope on RSV F. It consists of three univalent Nb017 molecules connected by a glycine-serine (GS) linker 87. In a phase 1/2a clinical trial, ALX-0171 inhaled daily for three days by 175 hospitalized RSV-infected children between 28 days and 24 months old was shown to be safe, as no serious treatment-related adverse events were found (NCT02309320). However, upon RSV infection develops in the lower respiratory tract, inhaling RSV therapeutic antibodies through the nose may not improve the clinical course of the disease 88. Using phage display library technology, Rossey *et al.* screened two candidate camelid single-domain antibodies (VHH), F-VHH-4 and F-VHH-L66, which have strong RSV-neutralizing activity (IC50 < 0.1 nM) 89. The results indicated that these antibodies performed similar to or better than F-specific mAbs D25 and AM22. F-VHH-4 and F-VHH-L66 inhibited RSV infection by blocking membrane fusion between the virus and the target Hep-2 cells* in vitro*. In addition, these VHHs also prevented RSV replication and lung infiltration caused by inflammatory monocytes and T cells in RSV-infected mice 89. These pre-F-specific VHHs represent promising anti-RSV drugs. Recently, Tiwari *et al.* presented a strategy of directly expressing neutralizing antibodies via mRNA in the lungs 90. When mRNA expressing palivizumab was delivered in the form of aerosols to the lungs of mice, the copy number of RSV F was reduced by approximately 90%. Moreover, RSV replication was significantly inhibited 7 days after an anchored VHH-expressing mRNA was transfected into the lungs of mice, and the antibody level persisted for at least 28 days. This method of expressing membrane-anchored broadly neutralizing antibodies in the lungs may be promising for preventing lung infection.

Although RSV G (298 amino acid residues) generally has highly variable and heterogeneous N- and O-glycosylated mucin-like regions, it also contains a CCR of ~ 40 amino acids. The CCR is not glycosylated but contains a CX3C chemokine motif; this motif can promote binding to the human chemokine receptor CX3CR1, which is a key step in RSV infection in human respiratory epithelial cells 91. Antibodies against the G protein are also in preclinical development, and these antibodies have been shown to inhibit virus attachment. Collarini *et al.* used a phenotypic cell-sorting technique to screen the high-affinity antibody 3G3, which could specifically bind to the CCR of the RSV G protein 92. This antibody showed good virus clearance activity in a mice model for infection prevention and treatment and is expected to be an attractive candidate for the clinical treatment of RSV. Anderson *et al.* obtained the mAb 131-2G against the RSV G protein using the hybridoma technique. The virus titer in the lungs of mice injected intraperitoneally with 300 μg of the antibody decreased by 99% 93. Lee *et al.* used the same method to screen two mAbs, 5H6 and 3A5. Intravenous injection of 100-200 μg of mAbs protected mice from RSV infection and promoted the clearance of the virus from the lungs 94. These antibodies may be used as alternatives to prevent RSV infection. However, the N-terminus and C-terminus of the extracellular domain of the G protein also contain multiple antibody-binding protected sites 95. Table 1 lists select potential RSV-neutralizing antibodies and related information.

## Vaccine

An effective vaccine to protect high-risk groups from severe RSV infection will be indispensable. In the 1960s, clinical trials of a formalin-inactivated RSV vaccine for infants aggravated RSV disease and eventually led to the death of two infants. This unacceptable situation may have been related to immune complex accumulation in the bronchioles induced by the vaccine with weak neutralizing activity but a high complement binding titer and the Th2-based CD4^+^ T cell response in the cellular immune response 97,98. Currently, dozens of promising vaccines are being developed to prevent RSV infection, and they are mainly attenuated vaccines, subunit vaccines and vector-based vaccines. The most fundamental principle of RSV vaccine design, regardless of type, always follows the principle that RSV-neutralizing antibodies need to be introduced into airway mucosa in the most reasonable way possible 99. Table 2 lists some representative vaccines and related information.

### Live attenuated vaccines

One of the basic goals of live attenuated vaccines is to limit the replication of RSV in recipients through traditional measures (such as heating or chemical treatment) or reverse genetic techniques to maintain an appropriate and natural balance of B and T cell responses, thereby avoiding vaccine-enhanced diseases. Currently, there are approximately 30 active clinical trials of live attenuated RSV vaccines registered at ClinicalTrials.gov.

Most of these vaccines are attenuated by deletion of genes such as those generated by M2, NS2/NS1 and L mutations. As mentioned above, deletions of RSV M2-2 or NS2/NS1 protein sequences have been shown to hinder or interfere with viral RNA replication. For example, both RSV MEDI ΔM2-2 and LID ΔM2-2 vaccines can induce a strong neutralizing antibody response to RSV 100,101. Some optimized versions carry two site mutations on the L gene, such as LID ∆M2-2/1030s, which achieved a better attenuated effect and higher RSV-neutralizing antibody titer than a maternal vaccine 102. Vaccines based on NS2/NS1 deletion, such as RSV ΔNS2 Δ1313 I1314L, RSV 6120/Δ NS2/1030s, and RSV 6120/ΔNS1, are under clinical evaluation (NCT01893554, NCT03916185 and NCT03387137). Recently, the vaccine D46/NS2/N/ΔM2-2-HindIII has been shown to have greater attenuated toxicity and a higher neutralizing antibody titer than the maternal vaccine LID ΔM2-2 (NCT03102034 and NCT03099291) 103. In addition, Meissa Vaccines produced the highly attenuated vaccine MV-012-968 by optimizing the codons of the NS2, NS1 and G genes, as well as by deleting the SH gene, which had been proven to have a strong protective effect in a cotton rat model. A phase 2 clinical trial for determining the efficacy of this vaccine in humans are currently being evaluated (NCT04227210). Recently, Jenkins *et al.* designed and produced the live attenuated RSV vaccine candidate rgRSV-L(G1857A)-G(L208A) 104 by introducing two modifications: one attenuating mutation in the L protein and another mutation in the G glycoprotein. The former mutation did not inhibit virus production in Vero cell culture but greatly reduced the transmission of the virus in human bronchial epithelium, and the latter mutation reduced the cleavage of the vaccine virus in Vero cells, thus increasing the yield and making the production more economical. In cotton rat treated with this candidate vaccine, RSV replication was undetectable even at a dose of 10^5^ plaque-forming units (PFU), and it was sufficient to completely protect against RSV A2 infection after 500 PFUs immunization 104. This may be a potential candidate for a live attenuated RSV vaccine.

### Protein/subunit vaccines

Currently, the protein/subunit vaccines evaluated in clinical trials are mainly focused on the F protein. For these vaccines, the focus is primarily on the epitopes that can induce a B cell response to produce strong neutralizing activity for generating vaccines with long-term protective effects. This kind of vaccine can minimize the production of non-neutralizing or weakly neutralizing antibodies in the process of immunization, thus weakening antibody-dependent enhancement (ADE) 105. RSV F DS-CaV1 is an F protein vaccine developed by NIAID based on the pre-F trimer DS-Cav1 with a stable conformation. In a phase 1 clinical trial, the evaluation of the safety and immunogenicity of DS-Cav1 in healthy adults aged 18-50 years showed that it was safe and induced strong RSV F-specific antibodies and neutralizing activity lasting more than 44 weeks (NCT03049488) 106. GlaxoSmithKline has produced two F-based vaccines, GSK3844766A and GSK3888550A, that have been subjected to phase 2 clinical evaluations for safety and immune response in the elderly and pregnant women, respectively. Both of these vaccines stimulated a strong immune response and were well tolerated 107. Currently, the company is preparing for the implementation of phase 3 clinical trials. Novartis RSV F protein nanoparticle, which is mainly based on the postfusion F conformation, shows antigenicity site II binding. It failed to protect newborns of vaccinated pregnant women in its phase 3 clinical trial 108. One of reason contributing to this clinical result may be due to the fact that the postfusion conformation cannot elicit a wider range of neutralizing antibodies as the prefusion conformation 109.

Phase 1 clinical trials have been completed on subunit vaccines, which are based on other proteins including DepoVax (DPX)-RSV and BARS13. DepoVax (DPX)-RSV, developed by Dalhousie University, is a candidate protein vaccine directed toward the extracellular domain of SH protein containing the lipid-based DepoVax agent. In a phase 1 clinical study with healthy adults aged 50-64 years, DPX-RSV showed good safety and a specific antibody response that lasted for more than one-half year (NCT02472548) 110. BARS13 is a protein vaccine developed by Advanced Vaccine Laboratories based on G protein, and a phase 1 clinical evaluation of its safety and efficacy in the elderly is being carried out (NCT04681833).

In addition, some promising studies have also provided a new path for the research and development of protein/subunit vaccines. Herv é *et al.* prepared a skin surface RSV vaccine using a Viaskin^®^ skin patch as the delivery platform and RSV N-nanorings (N) as subunit antigens 111. This needle-free vaccine may be better received by sensitive groups such as babies because it does not require skin preparation. In mice and pigs, it showed a high level of immunogenicity to promote a Th1/Th17-oriented immune response and provided strong protection against viral replication during RSV infection without aggravating clinical symptoms. A chimeric vaccine candidate, rBCG-N-HRSV, consisting of bacille Calmette-Guérin (BCG)-expressing RSV N, has been shown to have protective effects in mice. A single-dose vaccine can protect mice from pathogenic infection with 1×10^7^ PFUs of the RSV A2 clinical virus strain. A phase 1 clinical trial is currently under evaluation (NCT03213405) 112. In addition, Marcandalli *et al.* designed DS-Cav1 protein nanoparticles based on structural self-assembly 113. The nanoparticle scaffold I53-50 consists of 20 trimers and 12 pentamers for a total of 120 subunits. The two-component feature of this scaffold enables it to produce highly ordered, monodisperse immunogens. A stable pre-F DS-Cav1 trimer protein was presented on the outside of the nanoparticle substrate in the form of repeated arrays. When mice and nonhuman primates were inoculated, the immunogenicity of nanoparticles containing 20 DS-Cav1 trimers was 10-fold greater than that of the trimer DS-Cav1 alone 113. Most recently, Swanson *et al.* fused the RSV pre-F protein into self-assembled ferritin nanoparticles, which were modified with glycans to cover non-neutralizing or poorly neutralizing epitopes 114. Each of the optimized pre-F-NP contain 8 pre-F proteins. Pre-F-NP induced persistent pre-F-specific antibodies and produced effective neutralizing antibody responses in mouse and nonhuman primate models. Compared with the pre-F trimer DS-Cav1, pre-F-NP stimulated a stronger antibody response. These results encourage the continued development of these promising nano RSV vaccines.

### Vector-based vaccines

Vector-based vaccines transmit RSV antigens through vectors and induce specific immune responses. To date, eight vector-based vaccines for RSV infection have been tested in clinical trials, of which 4 (including MEDI-534, VXA-RSV-f oral, RSV001, Ad26.RSV.FA2) were ineffective or no longer under development. Ad26.RSV.Pre-F, developed by Janssen, is a vector-based vaccine based on human adenovirus 26 strain (Ad26) expressing stable pre-F protein 115. In a cotton rat model, the vaccine could induce the production of CD8^+^ T cells expressing IFN-γ and TNF-α markers of the Th1 immune response and showed minimal damage or inflammatory infiltration in the lungs after RSV challenge. Moreover, the high titer of RSV-neutralizing antibody produced after immunization could last for more than 30 weeks and produced protective immunity to RSV virus in the lung and nasal cavity. A phase 2a clinical trial conducted with 180 elderly patients older than 60 years of age showed that Ad26.RSV.preF had an acceptable safety profile with no observed interference of immune responses 116. Currently, the safety, responsiveness and immunogenicity of this vaccine in RSV-negative children aged 12-24 months are being evaluated in a phase 1/2 clinical trial (NCT03606512). Another clinical study evaluating the safety and immunogenicity of Ad26.RSV.preF in adults older than 60 years have not reported the results (NCT03502707). SeVRSV is a vaccine based on SeV, a mouse parainfluenza virus type 1 (PIV1), which encodes the full-length gene of RSV F 117. The safety and responsiveness of intranasal injection of the SeVRSV vaccine were evaluated in healthy adults aged 18-45 years in a phase 1 clinical trial. The study showed that the vaccine was well tolerated 118. ChAd155-RSV is an RSV vaccine developed by GlaxoSmithKline based on the design of chimpanzee adenovirus-155 (ChAd155) 119. In contrast to the previous two vector-based vaccines, this vector contains genes that encode three proteins (F, N and M2-1) of RSV. In a phase 1 clinical trial of 72 healthy adults aged 18-45 years, ChAd155-RSV was not found to cause significant safety issues, and its specific humoral and cellular immune responses were high 119. Currently, the results of two clinical trials with infants (a phase 2 study with infants aged 12 to 23 months (NCT02927873) and the phase 1 study with infants aged 6 and 7 months (NCT03636906)) have not yet been published. MVA-BN-RSV is a multivalent vector vaccine. The MVA-BN-based vector developed by Bavarian Nordic is a genetically engineered virus derived from the modified Ankara vaccinia virus (MVA)) that has lost the ability to replicate in human cell lines but can effectively infect mammalian cells 120, and it carries F, G, M2 and N genes encoding RSV structural proteins. The results of a phase 2 clinical evaluation of the dose and vaccination regimen of MVA-BN-RSV in adults older than 55 years showed that MVA-BN-RSV was safe and that a single dose could induce a cellular immune response biased towards Th1 cells and an antibody response that lasted for more than one-half year 121. Currently, a phase 3 clinical trial is being planned.

### mRNA-based vaccines

This strategy may not only eliminate the need for the purification of protein/subunit vaccines by fermentation, which is particularly challenging for antigens that are difficult to purify or that show poor stability, but may also prevent the anti-vector immune reaction encountered upon administration of vector-based vaccines. An mRNA vaccine can also induce strong humoral and cellular immune responses at the same time. In fact, data have shown that transcribed mRNA encapsulated in lipid nanoparticles (mRNA/LNP) is potentially safe and effective *in vitro* for preventing RSV diseases 122. mRNA-1345 is developed by Moderna encoding a prefusion F glycoprotein, which is conducting a phase I clinical study to assess the tolerance and responsiveness of young people, women of childbearing age, the elderly and seropositive children. The phase I mid-term results showed that there were no adverse events at a dose of 100 μg, and this vaccine produced RSV-neutralizing antibodies at a rate of more than 21-fold, compared with that of the control group, in the first month 123.

## Inhibitors

Ribavirin, a nucleoside analog, is the only clinically approved antiviral inhibitor for the treatment of RSV infection. However, the drug is restricted for the treatment of children because of concerns about teratogenicity and occupational exposure 124. At present, the development of small-molecule inhibitors of RSV infection is mainly based on two modes of action: in one mode, the invading virions are blocked from binding to the F protein on the surface of RSV, and in the other mode, the production of new virions is inhibited by blocking viral transcription and replication. Table 3 summarizes some representative inhibitors currently being developed for RSV infection and related clinical information.

GS-5806 (presatovir) is an RSV F inhibitor developed by Gilead Sciences. It can effectively prevent RSV F-mediated intercellular fusion, showing an average 50% effective concentration (EC50) of 0.43 nM for 75 different clinically isolated RSV A and B virus strains *in vitro* and low cytotoxicity in human cell lines and primary cell cultures 125. In a challenge study of intranasal RSV infection in healthy adults, participants treated with GS-5806 had a lower viral load and lower neutrophil count and total mucus weight than those in the control group, reducing the severity of clinical disease 126. A phase 2b clinical trial has been completed to evaluate the antiviral effect of GS-5806 on adult hospitalized patients with RSV infection, but no results have been published to date (NCT02135614). Another phase 2b study performed to evaluate the efficacy of presatovir in RSV LRTI in patients with hematopoietic cell transplants (HCTs) did not improve virological or clinical outcomes despite it being well tolerated 127. JNJ-53718678 (JNJ-678) is another effective RSV F protein inhibitor developed by Johnson Company. Similar to GS-5806, it blocks the invasion of virus particles by combining with pre-F with high affinity (Kd value of 7.4 nM). It showed strong anti-RSVA2 activity *in vitro*, and the EC50 value was 480 pM 128. Roymans *et al.* found that JNJ-53718678 could bind to the F protein with strong nanoaffinity activity. Oral treatment of newborn lambs with JNJ-53718678 effectively inhibited identifiable acute lower respiratory infections, even when treatment was delayed until external symptoms of RSV disease became apparent 129. A recent phase 2 clinical study of healthy adults showed that JNJ-53718678 treatment significantly reduced viral load and clinical disease severity (NCT02387606). RO-0529 and RV521, developed by Ark Biosciences and ReViral, respectively, have shown good anti-RSV clinical isolate activity *in vitro* and* in vivo*. Currently, RO-0529 is in phase 2/3 clinical trials for the evaluation of its efficacy in hospitalized adult and infant patients (NCT03699202 and NCT04231968). The phase 2a clinical results of RV521 showed that both 350 mg and 200 mg of the inhibitor could significantly reduce viral load and disease severity 5 days after RSV infection (NCT03258502) 128.

Another potentially interesting target is the N protein, which is an important component of the polymerase complex and the most conserved viral protein that plays an indispensable role in RNA transcription and replication. RSV604, an analog of 1,4-benzodiazepine, has been found to hinder optimal viral transcription by directly binding to RSV N 130. Some promising antiviral activities were shown in early clinical studies, but the studies were stopped because of its poor efficacy. Then, EDP-938 was identified after re-optimization of 1,4-benzodiazepine. The results showed that it inhibited RSV by blocking RSV N to prevent the virus from entering the postreplication stage of its life cycle. EDP-938 showed high antiviral activity against all RSV laboratory and clinical isolates *in vitro*, and its EC50 value was 21~64 nM 131. Currently, EDP-938 is being evaluated in a phase 2 clinical study (NCT04816721). ALN-RSV01, a small interfering RNA (siRNA), forms an RNA-induced silencing complex by targeting the mRNA of RSV N to inhibit translation and ultimately reduce viral load. In a phase 2 clinical study of 88 healthy adults, ALN-RSV01 significantly reduced the viral activity of participants with RSV infection 132. In another phase 2b clinical trial conducted with RSV-infected lung transplant patients, although ALN-RSV01 showed good tolerance and reduced the risk of bronchiolitis obliterans syndrome after RSV infection, there was no significant difference in viral parameters or symptom scores between the treatment group and the control group 133.

In addition, the large polymerase protein L of RSV is necessary for viral mRNA transcription and genome replication. The development of inhibitors for RSV L is also an attractive target for antiviral intervention. Currently, some potential inhibitors, such as lumicitabine (ALS-8176), AZ-27, PC786 and JNJ-64417184, are being evaluated in clinical trials. TMEM16A is a major secretory anion channel in airway epithelial cells that is upregulated during airway inflammation and asthma. Recently, Pearson *et al.* found that TMEM16A inhibitors showed the potential for preventing RSV infection 134. Thus, the effectiveness of TMEM16A inhibitors as anti-RSV drugs needs to be tested *in vivo* as they may provide new ways to develop RSV inhibitors. Most recently, Risso-Ballester *et al.* reported that A3E, a steroidal alkaloid cyclopamine analogue, can inhibit RSV replication by targeting RSV transcription factor M2-1 to dissociate and harden IB, and *in vivo* experiments demonstrated that A3E can significantly inhibit RSV infection in the lungs of mice 135.

## Conclusions and future perspectives

It has been more than 60 years since RSV was discovered, and RSV has become one of the most important pathogens causing respiratory diseases in the world. With continuous exploration, the roles of the various components of RSV are being explored in-depth, and the mysterious veil surrounding the mechanism by which RSV invades the host and its stepwise interaction with the host is being lifted. In the past decade, researchers have identified several key host cell receptors. In particular, the recent discovery of IGF1R, the main receptor of RSV infection in host cells, will undoubtedly stimulate many research ideas. Future experiments should be directed to determining how the F protein interacts with IGF1R at the atomic level. The elucidation of the structure of the F protein before and after fusion will undoubtedly play a guiding role in antibody development and vaccine design. Further study on the molecular structure that triggers the fusion of the F protein with the host membrane may stimulate new targets for the design of antibodies and small-molecule inhibitors. A clearer understanding of the molecular immune response of host cells in the process of RSV infection will also contribute to the design of new drugs targeting potential regulatory molecules in the host immune pathway, such as IFN-γ, IL-33, etc. Although no antibodies, vaccines or inhibitors have been approved, with the exception of palivizumab, there are approximately 30 clinical interventions being evaluated and a large number of preclinical candidates. In the next five to ten years, the clinical prevention and treatment of RSV infection can be highly anticipated. However, similar to other RNA viruses, surface glycoproteins are the prone to mutation. Therefore, for the development of antibodies and small-molecule inhibitors, the global mutations and distribution of RSV must be identified and monitored because they may affect the results of clinical trials and even the efficacy of future products. Therapy consisting of a cocktail of two or more antibodies or small-molecule inhibitors should also be considered because this type of synergistic approach may reduce viral escape from host immunity. Although combination therapy has been proven to be a key strategy for achieving synergy and preventing the development of drug resistance by other viruses, whether this strategy will be effective in RSV treatment remains to be determined. In addition, the timing of starting treatment may also be critical. For example, if anti-RSV treatment is used early in the course of infection, it is more likely to show clinical benefits. For vaccines, more clinical research on mRNA vaccine of RSV should be carried out. Current research and clinical progress data on the prevention of RSV infection are optimistic, and we believe that more than one effective program will be approved and marketed in the near future to improve our ability to control RSV infection.

## Figures and Tables

**Table 1:** Selected antibodies for the prevention of RSV infection

Prototypic antibodies	Target site	Method of generation	Reference
D25, MEDI8897 (Nirsevimab)	Antigen site Ø of the RSV pre-F protein	B cell culture	80,96
Palivizumab, motavizumab, MEDI-557	Antigenic site II of the RSV F protein	Hybridoma technology	76,77
Suptavumab (REGN-2222)	Antigenic site V of the RSV pre-F protein	VelocImmune technology	83,84
RB1, MK-1654	Antigenic site IV of the RSV pre-F protein	B cell culture	85
MPE8	Antigenic site III of the RSV pre-F protein	Single B cell cloning	86
ALX-0171	Antigenic site II of the RSV pre-F protein	Immune libraries of llamas	87
F-VHH-4, F-VHH-L66	The cavity in the intermediate region between antigenic site II and IV of the RSV F protein	Phage display libraries	89
3G3	CCR of the RSV G protein	B cell immunization	92
131-2G	CCR of the RSV G protein	Hybridoma technology	93
5H6 and 3A5	CCR of the RSV G protein	Hybridoma technology	94

**Table 2:** Selected RSV vaccines under clinical development

Types of vaccine	Vaccine name	Description	Clinical status	Clinical trial number
Live attenuated vaccine	RSV MEDI ΔM2-2	A M2-2 gene-deleted live attenuated vaccine	Phase 1	NCT01459198
	RSV LID ΔM2-2	A M2-2 gene-deleted live attenuated vaccine	Phase 1	NCT02952339; NCT02601612; NCT02237209
	LID/∆M2-2/1030s	An optimized M2-2 gene-deleted live attenuated vaccine	Phase 1	NCT02794870
	RSV ΔNS2 Δ1313 I1314L	An optimized NS2 gene-deleted live attenuated vaccine	Phase 1/2	NCT03227029; NCT03422237
	RSV 6120/∆NS2/1030s	An optimized NS2 gene-deleted live attenuated vaccine	Phase 1/2	NCT03387137
	RSV 6120/ΔNS1	An optimized NS1 gene-deleted live attenuated vaccine	Phase 1	NCT03596801
	D46/ns2/N/∆M2-2-HindIII	An optimized M2-2 gene-deleted live attenuated vaccine	Phase 1	NCT03099291; NCT03102034
Protein/subunit vaccine	VRC-RSVRGP084-00-VP	An F protein vaccine developed on the basis of the trimer pre-F protein DS-Cav1	Phase 1	NCT03049488
	GSK3844766A and GSK3888550A	An F protein vaccine developed on the basis of the F/pre-F protein	Phase 1/2	NCT04126213; NCT04138056; NCT04090658
	DepoVax (DPX)-RSV	An SH protein vaccine developed on the basis of lipid agent DepoVax	Phase 1	NCT02472548
	BARS13	A G protein vaccine	Phase 1	ACTRN12618000948291
Vector-based vaccine	Ad26.RSV.preF	Ad26 vector-encoding gene of the pre-F protein	Phase 2b	NCT03982199
	SeVRSV	SeV vector-encoding gene of the full-length F protein	Phase 1	NCT03473002
	GSK3389245A (ChAd155-RSV)	ChAd155 vector-encoding gene of the F, N and M2-1 proteins	Phase 1/2	NCT03636906; NCT02927873
	MVA-BN RSV	MVA-BN vector-encoding gene of the F, G, N and M2 proteins	Phase 2	EUCTR2017-004582-27-BE
mRNA-based vaccine	mRNA-1345	Encoding a prefusion F glycoprotein	Phase 1	NCT04528719

Ad26, adenovirus serotype 26; SeV, Sendai virus (also named mouse parainfluenza virus type 1); ChAd155, chimpanzee adenovirus-155; MVA-BN, modified Vaccinia Ankara from Bavarian Nordic. Sources: https://www.path.org/resources/rsv-and-mab-trial-tracker/?i=2682.

**Table 3:** Selected RSV vaccines under clinical development

Inhibitors	Target	Mechanism of action	Clinical status	Clinical trials
GS-5806 (Presatovir)	F protein	Targets the RSV F protein to inhibit the entry of the virus	Phase 2b	NCT02254421; EudraCT, #2014-002475-29
JNJ-53718678		Targets the RSV F protein to inhibit the entry of the virus	Phase 1b/2	NCT02593851; NCT03656510; NCT04056611
AK0529 (Ziresovir, RO-0529)		Targets the RSV F protein to inhibit the entry of the virus	Phase 2/3	NCT03699202; NCT04231968
RV521		Targets the RSV F protein to inhibit the entry of the virus	Phase 2a	NCT03258502
RSV604	N protein	Inhibits the interaction between the virus and host proteins that facilitate RSV604 binding of the N protein to block effective viral transcription.	Phase 1	NCT00416442
EDP-938		Targets binding of viral N protein to block RSV replication in the postreplication phase	Phase 2	NCT04816721
ALN-RSV01	RNA	Targets RSV N transcripts by forming an RNA-induced silencing complex to inhibit translation	Phase 2b	NCT01065935; NCT00658086
lumicitabine (ALS-8176)	Polymerase L	Targets RSV polymerase complex, causing chain termination of RNA synthesis	Phase 2a	NCT02673476
AZ-27		Inhibits the initiation of RNA synthesis from the promoter	Preclinical	Null
PC786		Interrupts RSV L-protein polymerase activity	Phase 1/2	NCT03382431
JNJ-64417184		Interrupts RSV L-protein polymerase activity	Phase 1	NCT04121052; NCT04258189

Sources: ClinicalTrials.gov and clinicaltrialsregister.eu.
